# Transcriptional Termination Enhances Protein Expression in Human Cells

**DOI:** 10.1016/j.molcel.2009.01.008

**Published:** 2009-02-13

**Authors:** Steven West, Nicholas J. Proudfoot

**Affiliations:** 1 Sir William Dunn School of Pathology, University of Oxford, South Parks Road, Oxford OX1 3RE, UK

## Abstract

Transcriptional termination of mammalian RNA polymerase II (Pol II) requires a poly(A) (pA) signal and, often, a downstream terminator sequence. Termination is triggered following recognition of the pA signal by Pol II and subsequent pre-mRNA cleavage, which occurs either at the pA site or in transcripts from terminator elements. Although this process has been extensively studied, it is generally considered inconsequential to the level of gene expression. However, our results demonstrate that termination acts as a driving force for optimal gene expression. We show that this effect is general but most dramatic where weak or noncanonical pA signals are present. We establish that termination of Pol II increases the efficiency of pre-mRNA processing that is completed posttranscriptionally. As such, transcripts escape from nuclear surveillance.

## Introduction

We recently presented a dissection of the events that lead to mammalian RNA polymerase II (Pol II) termination (West et al., 2008). During the termination process, the poly(A) (pA) signal is recognized by Pol II and transcripts are cleaved to present an uncapped substrate for 5′→3′ exonucleases (Kaneko et al., 2007; Kim et al., 2004; West et al., 2004). Degradation of the trailing transcript precedes termination, where the Pcf11 protein may play a key role (Luo et al., 2006; West and Proudfoot, 2008; Zhang and Gilmour, 2006). At least two closely related termination pathways exist that differ in the primary nascent RNA cleavage event, which depends on the downstream terminator (West et al., 2008). Some terminators act to pause Pol II, and in this case cleavage at the pA site acts as the 5′→3′ exonuclease entry site and precedes termination. Transcripts from other terminators, such as that downstream of the human β-globin gene, are cotranscriptionally cleaved directly over the terminator sequence (Dye and Proudfoot, 2001). In this case, pA site cleavage occurs on transcripts subsequently released from the template DNA after Pol II has terminated.

Many aspects of pre-mRNA processing and transcription are known to influence gene expression (Proudfoot et al., 2002). Transcripts are capped at their 5′ ends to protect them from 5′→3′ degradation; the splicing reaction competes with degradation pathways, which target unspliced transcripts (Bousquet-Antonelli et al., 2000); and 3′ end processing is important, because transcripts that fail to be cleaved and polyadenylated properly are retained in the nucleus and degraded (Hilleren et al., 2001; Milligan et al., 2005; Saguez et al., 2008; Vodala et al., 2008). The most obvious way that transcription impacts on gene expression levels is through the rate of Pol II initiation, which can be modulated by combinations of promoter or enhancer strength and *trans*-activating factors (Kadonaga, 2004). Elongation rate may also determine gene expression patterns by influencing alternative splicing (Cramer et al., 1997). Failed termination on a gene can impair the expression of downstream genes by reducing the accessibility of the promoter to Pol II (Greger et al., 2000). This process, known as transcriptional interference, is particularly relevant in lower eukaryotes, as they often have closely spaced genes. Whereas transcriptional interference reduces the expression of the downstream gene, failure to terminate transcription of the upstream gene is not thought to affect its own expression. Furthermore, it may be of little relevance in higher eukaryotes where most genes are positioned far apart. Significantly, we now show that transcriptional termination on a gene is required for its own optimal expression.

## Results

### The β-Globin Terminator Enhances Gene Expression

**Figure 1:**
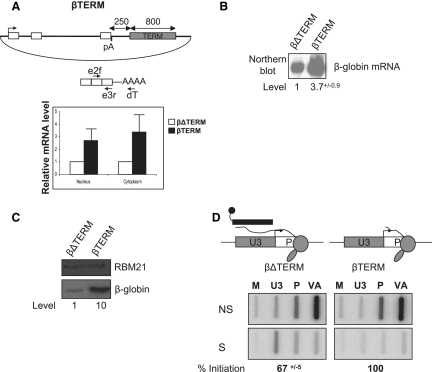
The β-Globin Terminator Enhances Gene Expression (A) The upper diagram shows βTERM. Promoter (arrow), exons (white box), pA signal (pA), and terminator (TERM) are shown. Distances between the pA site and terminator and the length of the terminator are shown. The lower diagram shows β-globin mRNA and the primers used for reverse transcription (dT) and real-time PCR (e2f/e3r) detection. The graph shows the relative level of nuclear and cytoplasmic β-globin mRNA from βΔTERM and βTERM samples, after equalizing to VA levels. mRNA levels were set at 1 for βΔTERM. Error bars show standard deviation from the mean (SDM). (B) Northern blot analysis of cytoplasmic RNA from βTERM and βΔTERM samples. mRNA levels are shown (βΔTERM is given a value of 1). SDM is shown. (C) Western blot analysis of HeLa cells transfected with βΔTERM or βTERM as well as the RBM21 expression construct. β-globin and RBM21 proteins are indicated. Quantitation is shown where βΔTERM is given a value of 1. (D) Hybrid selection NRO analysis of HeLa cells transfected with βΔTERM or βTERM as well as VA. Diagrams show U3 and P regions of the HIV promoter. The selection probe (black) selects U3 transcripts and promoter (P) transcripts that result from readthrough transcription (left diagram). P transcripts resulting from newly initiated Pol II are not selected (right diagram). The top data panels show transcripts not selected by probe (NS) and the lower panels show selected (S) transcripts. NRO M13 probes are shown above the relevant slot. Percent initiation is shown and given a value of 100% for βTERM. M is an empty M13 vector that shows background signal. SDM is shown.

To analyze the effect of termination on gene expression, we used the well-characterized human β-globin gene. Two plasmids were employed (Figure 1A): one containing the β-globin gene and its terminator sequence (βTERM) and one without the terminator (βΔTERM), the absence of which dramatically reduces termination efficiency (Dye and Proudfoot, 2001). In both cases, transcription is driven by the HIV promoter, transactivated by Tat. HeLa cells were transfected with βTERM or βΔTERM along with a cotransfection control plasmid encoding the adenovirus VA RNA. To measure β-globin mRNA expression, nuclear and cytoplasmic mRNA was analyzed using real-time PCR analysis (Figure 1A). In the presence of the terminator element (βTERM), levels of both nuclear and cytoplasmic β-globin mRNA were enhanced. We previously observed little difference in β-globin mRNA levels from these constructs, using probe-based RNA mapping techniques (Gromak et al., 2006). We suspect that β-globin mRNA was in excess of the probe level used in these experiments, and tested this assumption by using a large excess of probe to analyze β-globin mRNA by northern blot (Figure 1B). This experiment also revealed an enhancing effect of Pol II termination.

We next analyzed the effect of termination on the levels of β-globin protein. HeLa cells were transfected with βTERM or βΔTERM and a control plasmid expressing an HA-tagged protein (RBM21) which measures transfection efficiency. Following this, β-globin and RBM21 proteins were detected by western blotting (Figure 1C). Similar levels of RBM21 protein were detected in each case, demonstrating equal transfection efficiency. However, 10-fold more β-globin protein was detected in the βTERM protein sample as compared to the βΔTERM sample. We note that this is a greater effect than seen at the mRNA level, which may reflect additional translational regulation. These data indicate a positive function for termination in gene expression.

On βΔTERM, Pol II does not terminate efficiently and so may reduce new rounds of initiation (and gene expression) by reading around the plasmid and back into the promoter sequence, causing transcription interference. To measure interference, we performed hybrid selection nuclear run-on (NRO) analysis (Dye and Proudfoot, 1999) on HeLa cells transfected with βΔTERM and VA (Figure 1D). Radiolabeled nascent transcripts were hybridized to a biotinylated probe complementary to the U3 region of the HIV promoter. U3 is upstream of the promoter (P) region and therefore only transcribed by Pol II that fails to terminate and reads around the plasmid. Selection of the RNA hybrids with streptavidin-coated magnetic beads purifies transcripts continuous with the U3 region, which include P transcripts resulting from readthrough transcription. P transcripts deriving from new rounds of initiation will not be selected (see diagram). Selected transcripts (S) and those that escaped selection (NS) were hybridized to separate filters containing antisense M13 DNA probes. The majority of U3 signal was in the selected fraction, demonstrating efficient selection. Even so, most P signal was not selected, showing that it mainly derives from new rounds of initiation and not from readthrough transcription. Importantly, we controlled against RNA degradation as a cause for the appearance of P signal in the NS fraction (see Figure S1 available online). We repeated the experiment on βTERM transfected HeLa cells (Figure 1D) and no U3 transcripts were selected, presumably because termination prevents readthrough transcription into the U3 sequence. Quantitation of the P signals in the NS βTERM and βΔTERM fractions, relative to VA, revealed that transcription interference only reduces initiation to 67%. Consequently, only 33% of the P signal is due to transcriptional readthrough. This result was also confirmed by analysis of linear templates (Figure S2). Together, these data exclude the possibility that a transcriptional interference effect accounts for the changes in protein and mRNA levels.

### Enhancement of Gene Expression Is a General Function of Terminators

**Figure 2:**
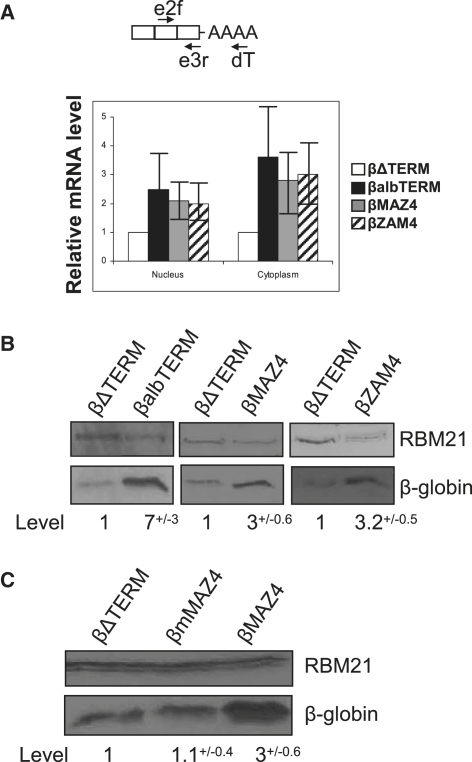
Gene Expression Is Enhanced by Different Terminators (A) RT-PCR analysis of nuclear and cytoplasmic β-globin mRNA from HeLa cells transfected with βΔTERM, βalbTERM, βMAZ4, or βZAM4. Primers are as in Figure 1A. The graph shows β-globin mRNA values (βΔTERM is set to 1). Error bars show SDM. (B) Western blot analysis of HeLa cells transfected with βΔTERM or βalbTERM, βΔTERM or βMAZ4, and βΔTERM or βZAM4 together with RBM21. β-globin and RBM21 proteins are indicated and quantitated. SDM is shown. (C) Western blot analysis of HeLa cells transfected with βΔTERM, βmMAZ4, or βMAZ4. β-globin and RBM21 proteins are indicated and quantitated. SDM is shown.

We next analyzed the effect of three other terminator elements on gene expression: the mouse serum albumin (MSA) terminator region (West et al., 2006b), the engineered MAZ4, and the reverse MAZ4 (ZAM4) (Gromak et al., 2006) sequences. MSA terminator transcripts are cotranscriptionally cleaved like those from the β-globin element (West et al., 2006b), whereas termination at MAZ4 and ZAM4 requires prior pA site cleavage (Gromak et al., 2006; West et al., 2008). These elements were cloned in place of the β-globin terminator to form βalbTERM, βMAZ4, and βZAM4, which were transfected into HeLa cells. Nuclear and cytoplasmic β-globin mRNA was then quantitated (Figure 2A). The presence of any of the three terminator elements significantly enhanced β-globin mRNA levels as compared to βΔTERM (Figure 2A). β-globin protein levels were also enhanced in each case (Figure 2B).

A termination-independent function for these sequences in pre-mRNA processing is unlikely. They share no apparent sequence homology, and the MAZ4 element has no effect on the processing of a synthetic pre-mRNA in vitro (Yonaha and Proudfoot, 1999). We therefore predict that their shared function in termination is responsible for enhanced gene expression. To test this more thoroughly, we employed a construct containing a mutated MAZ4 element (βmMAZ4) which is the same length as the MAZ4 element but does not promote Pol II termination (Gromak et al., 2006). We tested protein production from βmMAZ4, βMAZ4, and βΔTERM (Figure 2C). As before, ∼3-fold more β-globin protein was observed for βMAZ4 as compared to βΔTERM, whereas identical protein expression was observed for βΔTERM and βmMAZ4. Thus, consistent with its inability to promote Pol II termination, the mutant MAZ4 element does not enhance protein expression. This result reinforces the view that termination enhances gene expression.

### Termination Enhances Gene Expression Irrespective of the pA Signal Used

The other *cis*-acting sequence required for termination is the pA signal (Whitelaw and Proudfoot, 1986). It is generally thought that the rate of processing at the pA site correlates with termination efficiency and is a key determinant of gene expression levels (Edwalds-Gilbert et al., 1993). We explored the effects of the β-globin terminator element in the presence of pA signals that are processed relatively inefficiently. We replaced the β-globin pA signal in βTERM and βΔTERM with either the MSA or the human PMScl100 pA signal, forming ATERM, AΔTERM, PMTERM, and PMΔTERM. We have shown that the MSA pA signal is inefficient (West et al., 2006b) and that the PMScl100 pA signal contains an AUUAAA sequence instead of the consensus AAUAAA hexamer, which would be predicted to reduce its efficiency.

**Figure 3:**
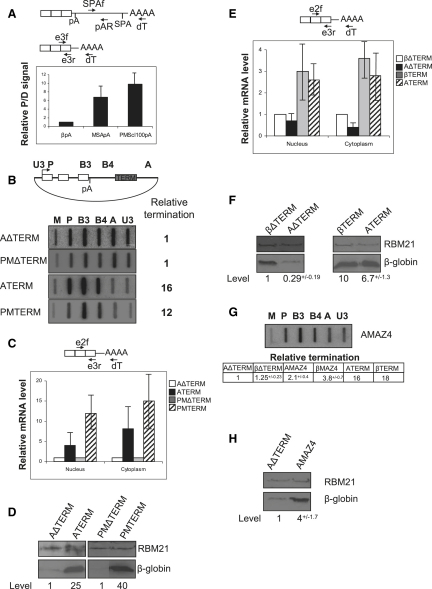
Termination Enhances Gene Expression Irrespective of the pA Signal Used (A) HeLa cells were transfected with constructs containing the β-globin (βpA), MSA (MSApA), or PMScl100 (PMScl100pA) pA signals upstream of the SPA. Diagrams show the primers used for reverse transcription (dT) and PCR of proximal (e3f/e3r) or distal (SPAf/pAR) signals. pA signal strength was determined as a ratio of mRNA processed at either the proximal (P) or distal (D) pA signal. βpA is given a value of 1. Error bars show SDM. (B) NRO analysis of HeLa cells transfected with AΔTERM, ATERM, PMΔTERM, or PMTERM. M13 probes are shown above the relevant slot and their position on the plasmid is shown in the diagram. Relative termination was quantitated by dividing the signal over readthrough probes A and U3 by those for probes P, B3, and B4. Values are set to 1 for AΔTERM and PMΔTERM. (C) RT-PCR analysis of nuclear and cytoplasmic β-globin mRNA from HeLa cells transfected with AΔTERM, ATERM, PMΔTERM, or PMTERM. Primers are as in Figure 1A. Values for the ΔTERM constructs are set at 1. Error bars show SDM. (D) Western blot analysis of β-globin protein in HeLa cells transfected with AΔTERM or ATERM (left blot) and PMΔTERM or PMTERM (right blot). β-globin and RBM21 control proteins are indicated and quantitated. (E) RT-PCR analysis of nuclear and cytoplasmic β-globin mRNA from HeLa cells transfected with βΔTERM, AΔTERM, βTERM, or ATERM. Primers are as in Figure 1A. Error bars show SDM. (F) Western blot analysis of β-globin and RBM21 proteins in HeLa cells transfected with βΔTERM, AΔTERM, βTERM, or ATERM. The lower panel on the left-hand side was exposed for longer, given the lower levels of β-globin present. SDM is shown. (G) NRO analysis of AMAZ4. M13 probe positions are shown in (B). The table shows quantitation (performed as for [B]) of the experiment and, for comparison, others on βTERM, βΔTERM, βMAZ4, AΔTERM, and ATERM. SDM is shown. (H) Western blot analysis of β-globin and RBM21 proteins in HeLa cells transfected with AΔTERM or AMAZ4. SDM is shown.

To compare the relative efficiencies of the β-globin, MSA, and PMScl100 pA signals, we performed a pA site competition assay on three constructs containing either of the three test pA signals followed by a synthetic pA signal (SPA) (Figure 3A). The SPA is only processed if the competing upstream site is relatively weak. HeLa cells were transfected with the three competition constructs (called βpA, MSApA, and PMScl100pA) and cytoplasmic RNA was isolated. Processing at the proximal test pA site and the distal SPA was then quantitated by RT-PCR. As compared to βSPA, 7- to 10-fold more processing at the SPA was observed when the MSA or the PMScl100 pA signal was positioned upstream. This result confirms our previous data demonstrating that MSA pA is weak (West et al., 2006b) and also shows that the PMScl100 pA signal is relatively weaker than the β-globin signal. Termination was then analyzed on ATERM, AΔTERM, PMTERM, and PMΔTERM using NRO (Figure 3B). As expected, this is inefficient on AΔTERM and PMΔTERM shown by the high signals over probes A and U3, which detect transcripts from Pol II that fails to terminate. Surprisingly, termination was very efficient on both ATERM and PMTERM, as shown by the low A and U3 signals. Thus, in this system, the β-globin terminator element promotes efficient termination irrespective of pA signal strength.

Next, we analyzed nuclear and cytoplasmic β-globin mRNA in HeLa cells transfected with ATERM, AΔTERM, PMTERM, or PMΔTERM (Figure 3C). We observed ∼4-fold more nuclear and ∼8-fold more cytoplasmic β-globin mRNA from ATERM as compared to AΔTERM and 10- to 15-fold more nuclear and cytoplasmic β-globin mRNA in PMTERM samples as compared to PMΔTERM samples. A substantial increase in protein levels was also observed (Figure 3D). These experiments reveal that termination can enhance gene expression even more in the presence of weak pA signals.

The effects of the β-globin terminator on gene expression from weak and strong pA signals were then directly compared. HeLa cells were transfected with βΔTERM, βTERM, AΔTERM, or ATERM and nuclear and cytoplasmic β-globin mRNA was analyzed (Figure 3E). Comparison of βΔTERM and AΔTERM samples revealed similar levels of nuclear mRNA, but levels of cytoplasmic mRNA were lower for AΔTERM, consistent with the weaker MSA pA. However, both nuclear and cytoplasmic levels of β-globin mRNA were similar in βTERM and ATERM samples, albeit much greater than with inefficient termination. Again, these results were confirmed at the protein level (Figure 3F). Thus, the β-globin terminator enhances gene expression from the MSA pA signal more than for the β-globin pA signal. As such, mRNA levels correlate more with termination efficiency than pA signal strength.

The above result was unexpected, because it has been suggested that gene expression correlates with pA signal strength (Edwalds-Gilbert et al., 1993). These data are rationalized by our recent observation that mammalian Pol II termination requires cleavage of nascent RNA, either within terminator transcripts or at the pA site (West et al., 2008). In the former case, pA site cleavage is not necessary for the termination process. Therefore, the inefficient MSA or PMScl100 pA signals do not reduce the efficiency of Pol II termination. This predicts that weak pA signals will only inhibit termination when pA site cleavage is solely responsible for nascent RNA cleavage, as we have shown for termination over MAZ4 (Gromak et al., 2006). These data are extended in Figure 3G, where the β-globin terminator in ATERM was replaced with the MAZ4 element and NRO analysis was performed. As predicted, the MAZ4 sequence promotes only partial termination beyond the weak MSA pA signal (Figure 3G). Nevertheless, termination on AMAZ4 is more efficient than on AΔTERM, and this still enhances protein expression (Figure 3H). It should be noted that hybrid selection NRO shows that the enhanced gene expression seen with weak pA signals cannot be accounted for by promoter interference (Figure S3).

### Termination Dramatically Enhances Erythropoietin Expression

The data so far show that Pol II termination is required for optimal β-globin expression. We next analyzed the effects of Pol II termination on the expression of a different human gene, erythropoietin (EPO). EPO does not contain a recognizable pA signal and instead uses an AAGAAC hexamer (Lin et al., 1985). This would not normally be expected to function, considering that single mutations in the AAUAAA hexamer are often enough to inactivate 3′ end processing (Sheets et al., 1990). Nevertheless, polyadenylated species were previously observed corresponding to use of this site (Lin et al., 1985), and we confirmed its use in our system by 3′ RACE (data not shown). Given the strong effects of termination on gene expression from weak pA signals, EPO provides a valuable further test of our findings.

**Figure 4:**
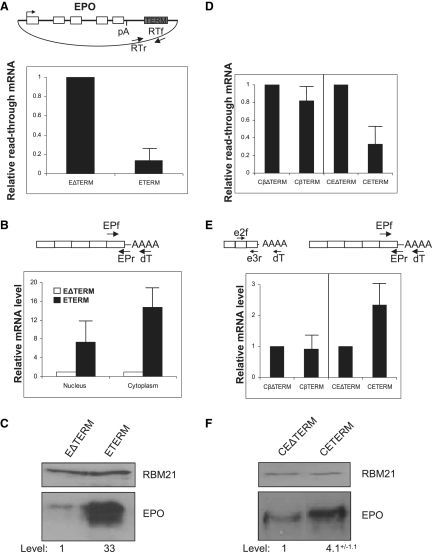
Termination Enhances Erythropoietin Expression (A) The diagram shows ETERM with nomenclature as for βTERM. The graph shows RT-PCR quantitation of readthrough RNA in HeLa cells transfected with EΔTERM or ETERM. The value for EΔTERM was set at 1. Primers used for reverse transcription (RTr) and PCR (RTf/RTr) are shown in the diagram. Error bars show SDM. (B) RT-PCR quantitation of nuclear and cytoplasmic EPO mRNA from HeLa cells transfected with EΔTERM or ETERM as well as VA. Values for EΔTERM are set at 1. The diagram shows primers used for reverse transcription (dT) and PCR (EPf/EPr). Error bars show SDM. (C) Western blot analysis of EPO protein secreted from HeLa cells transfected with EΔTERM or ETERM. EPO and the RBM21 control protein are indicated. Quantitation is shown (EΔTERM is given a value of 1). (D) RT-PCR quantitation of readthrough RNA from CβΔTERM, CβTERM, CEΔTERM, or CETERM. The ΔTERM samples are given a value of 1. Primers are as in (A). Error bars show SDM. (E) RT-PCR analysis of cytoplasmic β-globin and EPO mRNA in HeLa cells transfected with CβΔTERM, CβTERM, CEΔTERM, or CETERM. The ΔTERM samples are given a value of 1. The diagrams show primers for reverse transcription (dT) and PCR (e2f/e3r for β-globin and EPf/EPr for EPO). Error bars show SDM. (F) Western blot analysis of EPO protein from HeLa cells transfected with CEΔTERM or CETERM. EPO and the RBM21 control protein are indicated and quantitated. SDM is shown.

EPO was cloned into βΔTERM and βTERM in place of the human β-globin gene, still retaining the HIV promoter, forming EΔTERM and ETERM, respectively. These constructs and VA were transfected into HeLa cells and efficient termination on ETERM was confirmed by using a previously described RT-PCR assay that recapitulates NRO analysis (West and Proudfoot, 2008). Nuclear RNA was isolated and reverse transcribed with primer RTr, and cDNA was real-time PCR amplified using primers RTr and RTf to detect RNA beyond the terminator region (Figure 4A). We observed 10-fold less readthrough RNA for ETERM (indicative of efficient termination) as compared to EΔTERM.

We next analyzed nuclear and cytoplasmic EPO mRNA from HeLa cells transfected with EΔTERM or ETERM together with VA (Figure 4B). As for β-globin, we observed much higher levels of nuclear (8-fold) and cytoplasmic (15-fold) EPO mRNA in the ETERM sample as compared to EΔTERM. Levels of secreted EPO protein were also much greater (over 30-fold) for ETERM as compared to EΔTERM (Figure 4C). These data match our finding that termination dramatically enhances gene expression from weak pA signals and provides evidence that transcriptional termination is generally required for optimal gene expression.

### Termination Enhances Gene Expression Independent of the Promoter

We next investigated whether termination enhances gene expression in the context of a different promoter with distinct properties to the HIV promoter. The CMV promoter was chosen as it supports high levels of transcription but induces relatively slow elongation (Cramer et al., 1997). This contrasts with the HIV promoter, activated by Tat, which promotes highly processive Pol II elongation (Nogues et al., 2002; Parada and Roeder, 1996). We replaced the HIV promoter in βTERM, βΔTERM, ETERM, and EΔTERM with the CMV promoter, to form CβTERM, CβΔTERM, CETERM, and CEΔTERM, respectively. We analyzed termination efficiency on these constructs using RT-PCR to quantitate readthrough RNA (Figure 4D). There was little difference in the level of readthrough RNA between CβTERM and CβΔTERM, which indicates that termination is equally efficient on these constructs. However, we observed significantly less readthrough RNA from CETERM as compared to CEΔTERM, indicating a difference in termination efficiency. These data suggest that termination of the β-globin gene does not require a terminator when transcription is from the CMV promoter, but a terminator is still required to terminate transcription of the EPO gene. Because CMV-driven transcription is relatively nonprocessive, the strong β-globin pA signal is sufficient to induce termination without additional elements. In contrast, the noncanonical EPO pA signal still requires the terminator sequence for efficient termination.

If termination enhances gene expression, the above results predict that no difference in expression would be observed between CβTERM and CβΔTERM because the process is equally efficient on each construct. However, expression should be greater for CETERM than for CEΔTERM, because the terminator element improves termination on this construct. This was tested by transfecting HeLa cells with CβTERM, CβΔTERM, CETERM, or CEΔTERM and measuring cytoplasmic mRNA levels (Figure 4E). Little difference in β-globin mRNA levels was observed between the CβTERM and CβΔTERM samples, showing that the β-globin terminator does not further enhance gene expression if termination is already efficient. Crucially, 2.5-fold more EPO mRNA was recovered from the CETERM samples as compared to CEΔTERM, and a corresponding increase in EPO protein expression was observed (Figure 4F). These effects on EPO expression are not as great as those observed with the HIV promoter. However, this is in line with the fact that the difference in termination between CETERM and CEΔTERM is also less than that between ETERM and EΔTERM (compare Figures 4A and 4D). We conclude that, whereas a terminator is not always necessary for termination when transcription is driven by the CMV promoter, it remains the case that an enhanced termination process increases gene expression. This important observation shows that increasing termination enhances gene expression irrespective of the promoter.

### Mechanism of Increased Gene Expression by Efficient Pol II Termination

We sought to establish why termination enhances gene expression. Figure 1D shows that inefficient termination only reduces transcription initiation efficiency, through interference effects, by 33%. Yet, we demonstrate a significantly larger reduction of gene expression, implying that synthesized transcripts are degraded before they are translated. The surveillance mechanism is likely to be a nuclear process because nuclear mRNA levels are affected.

**Figure 5:**
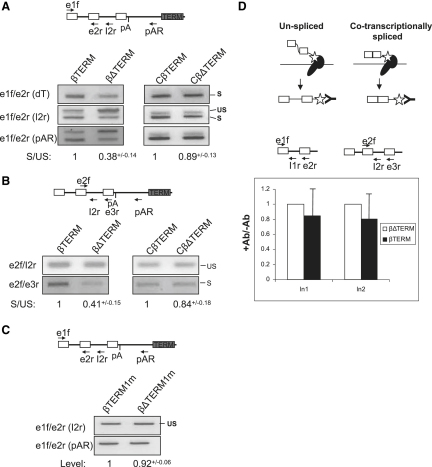
Termination Enhances Pre-mRNA Splicing (A) RT-PCR analysis of intron 1 splicing in HeLa cells transfected with βTERM, βΔTERM, CβTERM, or CβΔTERM. The diagram shows positions of the primers used in this experiment. The primer used for cDNA synthesis is indicated in brackets beside each panel with the PCR primer pair indicated to its left. Unspliced (US) and spliced (S) products are indicated. Real-time PCR quantitation of the ratio of spliced to unspliced (S/US) is shown. SDM is shown. (B) RT-PCR analysis of intron 2 in HeLa cells transfected with βTERM, βΔTERM, CβTERM, or CβΔTERM. Primers are indicated as in (A). It should be noted that different cycle numbers were used for the separate PCR reactions. Real-time PCR quantitation of the ratio of spliced to unspliced (S/US) is shown. SDM is shown. (C) RT-PCR analysis of pre-mRNA stability in HeLa cells transfected with βTERMm1 or βΔTERMm1. Primers are indicated as in (A). Real-time PCR quantitation is shown. SDM is shown. (D) BrUNRO analysis of cotranscriptional splicing. The top diagrams show the procedure, where immunoprecipitation of brU (star) detects cotranscriptional splicing (right) or introns that are not spliced during transcription (left). The lower diagrams show the primers used for reverse transcription (e2r and e3r) and PCR (e1f/I1r and e2f/I2r) to detect intron 1 and 2, respectively. Quantitation shows the signal (set at 1 for βΔTERM) obtained after subtracting the minus antibody control value. Error bars show SDM.

It is well established that Pol II transcription and pre-mRNA processing are coupled (Proudfoot et al., 2002). Because removal of terminator elements inhibits gene expression, we tested whether pre-mRNA splicing efficiency is also reduced. β-globin pre-mRNA splicing was analyzed in HeLa cells transfected with βTERM and βΔTERM (Figure 5A). To be confident that changes are due to termination, we also analyzed CβTERM and CβΔTERM, which share the same sequence differences as βTERM and βΔTERM but support similar levels of termination (Figure 4D). Nuclear RNA was reverse transcribed with dT to detect cleaved and polyadenylated transcripts, with primer I2r to detect unspliced transcripts or with pAR to detect transcripts not yet cleaved at the pA site. These cDNAs were amplified with primers e1f and e2r to detect spliced (S) and unspliced (US) RNA (Figure 5A). For the dT primed cDNA, only spliced RNA was detected in each case, indicating that the majority of cleaved and polyadenylated transcripts are also spliced. We next amplified the I2r and pAR cDNA with primers e1f and e2r to analyze the splicing status of pre-mRNAs. A higher ratio of spliced to unspliced transcripts was recovered from βTERM samples as compared to βΔTERM. Because the ratio of spliced and unspliced transcripts was similar for CβTERM and CβΔTERM, which both terminate equivalently, termination is likely to account for splicing differences between βTERM and βΔTERM. We next analyzed splicing of intron 2 using the same RNA samples. Because intron 2-retaining pre-mRNAs are more than 1 kilobase larger than spliced transcripts, primers spanning this region are susceptible to PCR competition. To circumvent this, intron 2-retaining and spliced transcripts were detected from the same pAR primed cDNA, using separate PCR primer pairs: e2f/e3r and e2f/I2r to detect spliced and unspliced transcripts, respectively (Figure 5B). There was a higher ratio of spliced to unspliced transcripts for βTERM as compared to βΔTERM. Again, a similar ratio was observed for CβTERM and CβΔTERM. We conclude that termination enhances splicing of both β-globin gene introns.

A potential criticism of the above result could be that pre-mRNA is degraded in the βTERM sample more efficiently than for βΔTERM. This is unlikely, given the lack of change between CβTERM and CβΔTERM samples. Even so, we repeated our analysis on a further two constructs (βΔTERM1m and βTERM1m) which contain a mutated first intron (Figure 5C). This mutation prevents splicing but not termination (Dye and Proudfoot, 1999), and so allows us to look at differences in the stability of the two pre-mRNAs. Only unspliced transcripts were observed in the analysis and the abundance of pAR and I2r primed cDNAs was unchanged, showing that these pre-mRNAs do not have significantly different stabilities.

### The Effect of Termination on Pre-mRNA Splicing Is Posttranscriptional

The enhanced splicing as a result of termination suggests a posttranscriptional effect. We therefore analyzed cotranscriptional splicing on βTERM and βΔTERM using a modified NRO protocol to incorporate bromo-labeled UTP (brU) into nascent RNAs which were purified using a brU-specific antibody (Lin et al., 2008). We purified brU-labeled RNA from HeLa cells transfected with βTERM or βΔTERM and examined the levels of transcripts containing intron 1 and intron 2 (Figure 5D). Cotranscriptional splicing is expected to reduce the level of intron-containing RNAs that are recovered. cDNA was synthesized with primers e2r or e3r and PCR amplification was with the e1f/I1r or e2f/I2r primer pairs to detect intron 1 and intron 2, respectively. After subtracting the background, obtained from minus antibody controls, we observed little difference in the levels of intron 1 and intron 2 between the βTERM and βΔTERM samples. We also directly analyzed spliced exons 2 and 3 but recovered no above-background signal (data not shown and Figure S4). These data reveal little difference in the cotranscriptional splicing of βTERM and βΔTERM transcripts. The difference in the levels of spliced transcripts observed in total nuclear βTERM and βΔTERM samples is therefore likely to reflect posttranscriptional splicing as a result of termination.

### The Exosome Degrades Some Transcripts When Termination Is Inefficient

**Figure 6:**
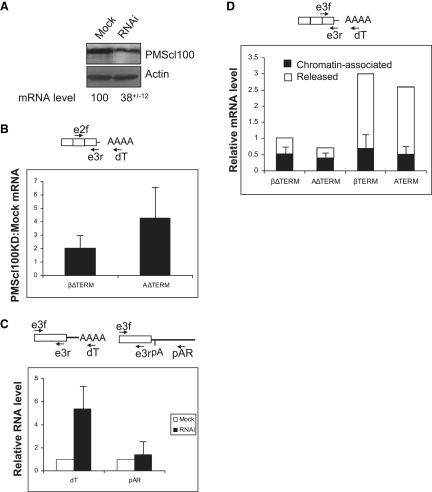
The Exosome Degrades Transcripts When Termination Is Inefficient (A) Western blot analysis of PMScl100 and actin proteins from mock treated and PMScl100-depleted HeLa cells. Quantitation of PMScl100 mRNA is shown underneath. SDM is shown. (B) Analysis of cytoplasmic β-globin mRNA in mock treated or PMScl100-depleted HeLa cells transfected with βΔTERM or AΔTERM. The graph shows the fold increase in cytoplasmic mRNA in PMScl100-depleted cells as compared to mock treated cells. Error bars show SDM. (C) RT-PCR analysis of nuclear mRNA and pre-mRNA in HeLa cells transfected with AΔTERM. The diagrams show the target species (3′ end processed RNA on the left and RNA, not cleaved at the pA site, on the right). Primers used for reverse transcription (pAR and dT) and PCR (e3f/e3r) are also shown. The graph shows relative RNA levels (the value for mock treated cells is 1). Error bars show SDM. (D) RT-PCR analysis of chromatin-associated (black) and released (white) nuclear β-globin mRNA isolated from HeLa cells transfected with βΔTERM, AΔTERM, βTERM, or ATERM. Primers are as in Figure 1A. The proportion of mRNA in each fraction is shown superimposed onto the levels of total nuclear β-globin mRNA obtained from the experiment shown in Figure 3E. Error bars show SDM.

We next asked what degrades the transcripts when termination is inefficient. To this end, we depleted the nuclear exosome subunit PMScl100 using RNA interference (RNAi). Western blot analysis of PMScl100 protein in cells that had been mock treated or transfected with PMScl100-specific siRNAs showed that levels were depleted by 2- to 3-fold (Figure 6A). Equal levels of actin were observed showing that loading was equivalent. These data were substantiated by quantitative RT-PCR analysis of PMScl100 mRNA, which was reduced to 38%. We observed a similar effect with a further two PMscl100-specific short hairpin RNAs (data not shown), which also resulted in similar phenotypes to those described below.

The effect of this depletion was tested in situations where termination and splicing are inefficient and for strong and weak pA signals. Mock and PMScl100-depleted cells were transfected with βΔTERM or AΔTERM and levels of cytoplasmic β-globin mRNA were analyzed by RT-PCR (Figure 6B). We observed increased levels of cytoplasmic mRNA in PMScl100-depleted cells as compared to mock treated cells, identifying PMScl100 as part of the mechanism that suppresses gene expression when termination is inefficient. Interestingly, the effect of exosome depletion was greater for AΔTERM than for βΔTERM. This is in line with our finding that termination enhances gene expression to a greater degree for weaker pA signals. PMScl100 depletion has little effect on βTERM mRNA levels (West et al., 2006a), which is consistent with the termination process reducing the susceptibility of transcripts to degradation. Depletion of the cytoplasmic exonuclease Xrn1 had little effect on mRNA levels (data not shown), confirming a nuclear surveillance process.

We next determined the timing of degradation in relation to 3′ end processing. Mock and PMScl100-depleted cells were transfected with AΔTERM, and RNA samples were reverse transcribed with pAR (to detect uncleaved) or dT (to detect cleaved and polyadenylated RNAs). Subsequent PCR was with the e3f and e3r primer pair (Figure 6C). As before, PMScl100 depletion substantially increased the level of RNA cleaved at the pA site (dT primed). However, there was much less of an effect on uncleaved transcripts. These data show that PMScl100 targets AΔTERM transcripts for degradation after cleavage at the pA site. Presumably, the exosome requires free RNA termini to degrade a transcript. For AΔTERM, and other cases where there is no terminator transcript cleavage, this is primarily provided by pA site cleavage. Where terminator transcripts are cleaved, we have shown this to provide additional targets for the exosome (West et al., 2006a).

Collectively, the above results show that inefficient termination promotes the degradation of transcripts by the exosome after pA site cleavage. These data predict that termination releases mRNA from this decay, which can exist close to transcription sites (Hilleren et al., 2001). We finally employed a technique that separates chromatin-associated transcripts from those released into the nucleoplasm (West et al., 2008). We included constructs with either strong or weak pA signals in the analysis. Nuclei were isolated from HeLa cells transfected with βΔTERM, AΔTERM, βTERM, or ATERM, fractionated into chromatin-associated and released fractions, and analyzed using RT-PCR (Figure 6D). RNA was reverse transcribed with dT and then real-time PCR amplified with primers e3f and e3r to detect all polyadenylated species, both spliced and unspliced. Similar levels of chromatin-associated polyadenylated RNA were recovered in each case (black section of bar) but much more released polyadenylated RNA was present in the βTERM and ATERM samples (white section of bar). This again suggests that release of transcripts by termination enhances gene expression. The relevance of this observation to the enhanced gene expression is emphasized by the close correlation between the levels of released polyadenylated RNA and cytoplasmic mRNA (compare Figures 6D and 3E).

## Discussion

The data presented in this study show that termination releases transcripts from the DNA template, which promotes their processing and subsequent gene expression. Previous studies identified the pA signal as a key determinant of gene expression levels and termination efficiency (Edwalds-Gilbert et al., 1993). Although consistent with these data, we show that weak pA signals have no effect on the termination process when the β-globin terminator is used in conjunction with the HIV promoter. This is presumably because cleavage of terminator transcripts obviates the need to cleave at the pA site by enabling termination to occur irrespective of pA signal strength. When termination is reliant on pA site cleavage, the strength of the pA signal determines its efficiency (Edwalds-Gilbert et al., 1993; Gromak et al., 2006; Orozco et al., 2002).

Our studies show that the CMV promoter reduces the necessity for terminator elements, as termination on CβTERM and CβΔTERM is equivalent. However, a terminator element is still required to terminate transcription of the EPO gene when the CMV promoter is used. This could be accounted for by the differences in the EPO and β-globin pA signals. The strong β-globin pA signal is rapidly processed to drive efficient pA site-dependent termination on CβΔTERM. However, the EPO pA signal is not sufficiently strong to do the same on CEΔTERM so that, in this case, efficient Pol II termination depends on the terminator. A strong pA signal may permit Pol II termination to occur when transcription initiates from the CMV promoter, if elongation is slow enough to negate the need for a pause element. Such pause sites are normally needed for pA site cleavage-dependent termination when transcription is from the HIV promoter. In support of this being the case, CMV transcription promotes splicing patterns that are typical of slow elongation (Cramer et al., 1997). Furthermore, a higher percentage of β-globin exons are cotranscriptionally spliced when transcription is driven by the CMV promoter, as compared to when it is from the HIV promoter (Figure S4). These data suggest that Pol II is less processive when initiating at the CMV as compared to the HIV promoter, which is known to promote highly efficient elongation (Nogues et al., 2002; Parada and Roeder, 1996).

Our results show that a variety of unspliced pre-mRNA accumulates when Pol II termination is inefficient, suggesting an influence of termination on pre-mRNA processing. Data in Figure 5 suggest that many introns may not be removed until after termination. Indeed, it has been shown that artificial release of pre-mRNA from transcription sites can have a positive effect on splicing of β-globin gene transcripts (Bird et al., 2005). We have also shown that 3′ end processing, another event with a robust connection to transcription, can also occur posttranscriptionally (West et al., 2008). The present study shows that when these processes occur after termination, the transcripts are less susceptible to exosome degradation. Effectively, termination promotes escape from a nuclear surveillance process by releasing transcripts from sites of synthesis, a theory substantiated by our finding of high levels of released transcripts upon termination (Figure 6D). Further evidence that termination may be involved in mRNA release comes from experiments in yeast which identify 3′ end formation signals as important mediators of RNA tethering to transcription sites (Abruzzi et al., 2006). Unlike the situation in mammals, 3′ end formation signals are often the sole termination sequences in yeast.

**Figure 7:**
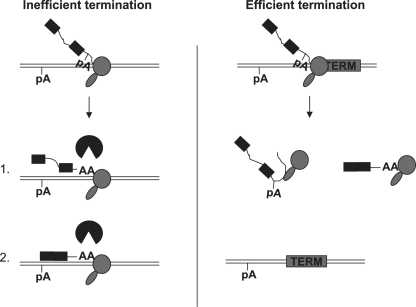
Model (Left) When termination is inefficient, pre-mRNA splicing is compromised and polyadenylated RNA species (1 and 2) are retained at transcription sites. These events are surveyed by the nuclear exosome, which degrades aberrant RNA and reduces gene expression. (Right) Termination enhances gene expression by promoting more efficient pre-mRNA processing and so reducing the susceptibility of transcripts to degradation.

We show in these studies that gene expression can be greatly enhanced by transcriptional termination. As indicated in the model (Figure 7), we predict that Pol II release from the DNA template facilitates pre-mRNA processing and further allows pre-mRNA/mRNA to escape surveillance. Our findings may have wide-ranging implications for in vivo protein production. This may be especially true in situations where efficient protein expression is difficult or expensive to achieve. Efficient termination may also be required to optimize gene delivery for gene therapy applications. It seems likely that Pol II termination represents a key stage in maintaining high levels of gene expression.

## Experimental Procedures

### Nuclear Run-On Analysis

NRO and hybrid selection NRO have previously been described (Ashe et al., 1997; Dye and Proudfoot, 1999). M13 probes were P, U3, and VA (Dye and Proudfoot, 1999), B3 and B4 (Ashe et al., 1997), and A (West et al., 2004). Template U3 selection probe was made by inserting an AvaI/PvuII restriction fragment, from βTERM, into pGEM4, and the upstream U3 probe was made by PCR amplification of βΔTERM with primers U35′ and U3T7. These clones were transcribed by SP6 and T7 polymerase (Dye and Proudfoot, 1999). The brUNRO protocol was performed as described (Lin et al., 2008).

### RT-PCR

cDNA was made using SuperScript III (Invitrogen), and 1 μl of the 20 μl reaction was analyzed by real-time PCR (10 pmol of each oligo, 1 μl of cDNA, 7.5 μl of SYBR green mix [QIAGEN], and water to a final volume of 15 μl) or semiquantitative PCR (Taq polymerase, Bioline) (1 μl of 10 mM dNTPs, 10 pmol of each primer, 1.5 mM magnesium chloride, 1× manufacturer's buffer). Experiments were quantitated after subtraction of values obtained from minus RT samples.

### Western Blotting

Fifty percent of lysate from a confluent 5 cm dish of HeLa cells was used for analysis. For secreted EPO, 10–100 μl of culture media was used. Membranes were probed with anti-human β-globin (Santa Cruz Biotechnology) at 1:1000, anti-EPO (Santa Cruz Biotechnology) at 1:1000, anti-PMScl100 (Abcam) at 1:1000, and anti-actin (Sigma) at 1:1000 or anti-HA (Santa Cruz Biotechnology) at 1:1000. Secondary antibodies were anti-mouse (Sigma) at 1:2000 or anti-rabbit (Sigma) at 1:2000. Signals were detected with an ECL kit (GE Healthcare) and quantitated using ImageQuant software.

### Northern Blotting

The protocol is available at http://www.narrykim.org/Northern_blot_analysis_for_microRNA.pdf. RNA samples were RNase H cleaved using primer 4.5 and dT. RNA was fractionated on a 6% gel and products were detected using 5′ ^32^P-labeled e3r primer.

### Transfections

Semiconfluent HeLa cells, in 5 or 10 cm plates, were transfected with 1–5 μg of reporter plasmid, 1–2 μg of VA plasmid, and 1.5 μg of Tat plasmid. Lipofectamine 2000 (Invitrogen) was used.

### RNA Isolation

The procedures for isolating nuclear and cytoplasmic RNA (West and Proudfoot, 2008) and for separating nuclear RNA into chromatin-associated and released fractions (Dye et al., 2006) have been described.

### Plasmids

Tat (Adams et al., 1988), VA (Dye and Proudfoot, 1999), and βTERM and βΔTERM (previously called βΔ5-7 and βΔ5-10) (Dye and Proudfoot, 2001); βMAZ4, βZAM4, and βmMAZ4 (previously called pMAZ4, pZAM4, and pmMAZ4) (Gromak et al., 2006); and AΔTERM and βalbTERM (previously called βΔ5-10ApA and βalb) plasmids (West et al., 2006b) have all been described. ATERM was made by inserting a TERM5′/TERM3′ PCR product into a vector prepared by PCR amplification of βΔ5-10ApA using APR/RTf primers. PMΔTERM and PMTERM were made by inserting a PCR product, generated by PMF/PMR amplification of HeLa cell DNA, into vectors prepared by F/e3 PCR amplification of βΔTERM or βTERM, respectively. AMAZ4 was made by inserting an APF/APR PCR product into a vector generated by PCR amplification of βMAZ4 with the F/e3r primer pair. The EPO gene was amplified from HeLa cell genomic DNA using primers E5′/E3′. EΔTERM was made by inserting EPO into a vector prepared by PCR amplification of βΔTERM with primers TAR3′ and RTf. ETERM was made by inserting EPO into a vector prepared by PCR amplification of βTERM using primers TAR3′ and TERM5′. The RBM21 expression plasmid was a kind gift from Chris Norbury. RBM21 is a member of the recently discovered family of noncanonical poly(A) polymerases. The βpA and MSA competition clones are described elsewhere (West et al., 2006b). The PMScl100pA competition clone was made by inserting a PMF/PMR PCR product into a vector made by PCR amplification of the βpA competition clone using primers SPAf and e3. For the CMV promoter constructs, the HIV promoter was removed by an AvaI/AflII digest and the CMV promoter, obtained by BglII/HindIII digest of pcDNA3.1 (Invitrogen), was inserted. βTERMm1 and βΔTERMm1 were made by ligating a PCR product generated by pAR/TERM5′ and pAR/RTf amplification of HIVβIVS1SAmut (Dye and Proudfoot, 1999).

### Nuclear RNA Fractionation

This protocol was previously described (Dye et al., 2006).

### RNA Interference

RNA interference of PMScl100 was previously described (West et al., 2006a).

### Primers

Primer sequences are in Table S1.

## Supplemental Data

Document S1. Four Figures and One Table
