# Transcriptional Termination Enhances Protein Expression in Human Cells

**DOI:** 10.1016/j.molcel.2016.01.021

**Published:** 2016-02-04

**Authors:** Steven West, Nicholas J. Proudfoot

(Molecular Cell *33*, 354–364; February 13, 2009)

In Figure 3F of the above article, we mistakenly used an exposure of the RBM21 control from βΔTERM and AΔTERM (left panel) for the βTERM and ATERM samples (right panel). The corrected figure appears below. We would also like to clarify that, in Figures 1B, 1C, 2B, 3D, 4F, and 6A, we removed irrelevant intervening lanes as was common practice. However, we did not indicate this in the figure legends as we should have. We recently provided the journal with full original images for these experiments as confirmation. Our oversights do not influence the conclusions of the paper. We apologize for any problems that we may have caused.

**Figure 3F:**
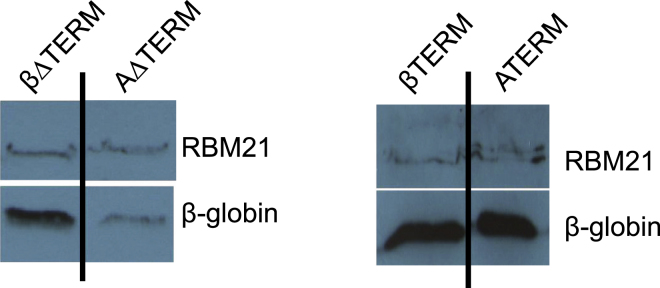
Termination Enhances Gene Expression Irrespective of the pA Signal Used

