# Supplementary material for: Transcriptional Termination Enhances Protein Expression in Human Cells
Source: Mol Cell. 2009 Feb 13;33(3-9):354–64. doi: 10.1016/j.molcel.2009.01.008 (PMC2706331; doi:10.1016/j.molcel.2009.01.008)

Molecular Cell, Volume *33*

Supplemental Data

Transcriptional Termination Enhances Protein Expression in Human Cells

Steven West and Nicholas J. Proudfoot

Table S1. Oligonucleotides (5'→3')

e2f:	TGGCCTGGCTCACCTGGACAACC
e3r:	ATCCAGATGCTCAAGGCCC
RTf:	CAGGAAACTATTACTCAAAGGGTA
pAR:	CTTGAATCCTTTTCTGAGGGATG
RTr:	AGAAAATACCGCATCAGGCGCCAT
TAR3'	GAGCTTTATTGAGGCTTAAGCAG
TERM5'	GCATAGTGTTACCATCAACCA
TERM3'	TTTCCTGATTCTCCCACCCCC
PMF	GCTTCAGGTACAACTGGCCAC
PMR	GGAGCACACTCACCTGCCCAC
EPf'	AAGCTGTGACTTCTCCAGGTC
EPr'	TGGTTTCAGTTCTTGTCAATG
E5'	ATGGGGGTGCACGGTGAGTAC
E3'	TCAGACAGGCTGTGTGAGACAG
APR	AAAGGCAGGGATTCCTCTGAGCC
APF	CCCTAAGGAACACAAATTTCTTTA
pAF	CCACAAGTATCACTAAGCTCGC
R	AACTAGCTCTTCATTTCTTTATG
F	CCTTGGGAAAATACACTATATC
4.5	TTGTGGGCCAGGGCATTAGCCACA
SPAf	CCTTGGGAAAATACACTATATC
I2r	CTATGACATGAACTTAACCATAG
e1f	ACTCCTGAGGAGAAGTCTGCC
e2r	TTTCTTGCCATGACCCTTCACC
I1r	TCAGTGCCTATCAGAAACCC
e3f	CCACAAGTATCACTAAGCTCGC
U35'	TTACGGTTCCTGGCCTTTTGCTGG
U3T7	TAATACGACTCACTATAGGGAGGTTTCCTGTGTGAAATTGTTATCCGC

Figure S1: RNA degradation does not influence interpretation of the hybrid selection NROs.

To control for degradation being responsible for the reduced P signal in Figure 1D, the hybrid selection NRO experiment was repeated on βΔTERM but the position of the selection probe was altered. This time, transcripts upstream of U3 were selected (see left diagram). If RNA degradation were responsible for the reduced P signal then few U3 transcripts will be selectable using this upstream probe. However, we were still able to select as many U3 transcripts with the upstream probe as were selected with a probe complementary to U3 itself. Less than 40% of P transcripts were co-selected consistent with initiation only being reduced to around 67% (seen in Figure 1D). These data indicate that RNA degradation does not prevent the selection of P transcripts.

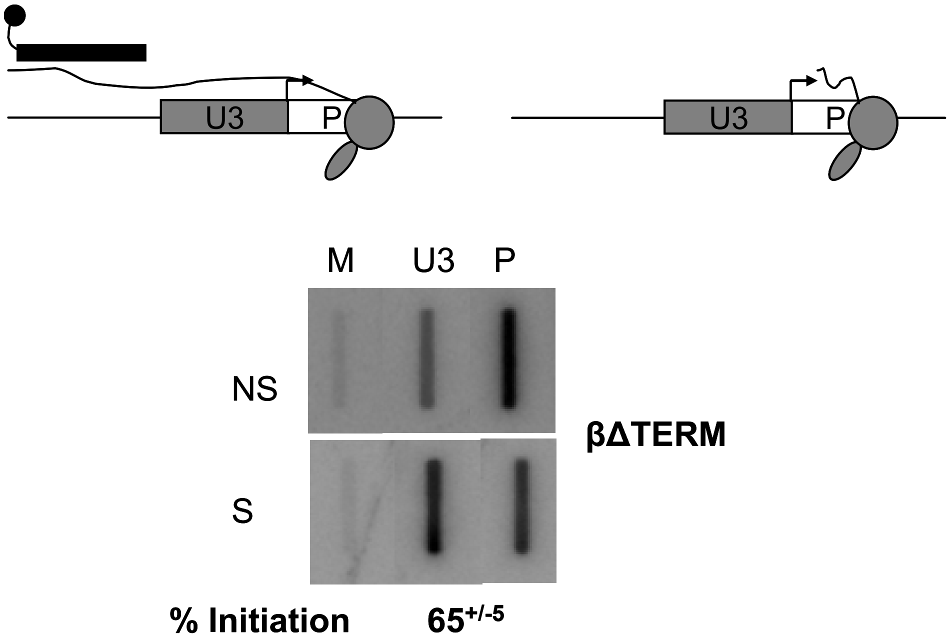

Figure S2: Analysis of mRNA levels from linear templates

ATERM and AΔTERM were linearised (top two diagrams) by restriction digestion upstream of the promoter (refer to Figure 3 for description of these plasmids). These constructs, along with the VA control plasmid, were transfected into HeLa cells and nuclear levels of β-globin mRNA were analysed by real-time RT-PCR. Lower diagram shows the primers used for reverse transcription (dT) and PCR (e2f/e3r). We observed ~2.5 fold higher levels in ATERM samples as compared to AΔTERM samples. In Figure 3C, an experiment on the same circular templates revealed a 3.8 fold difference in nuclear mRNA levels. The figure of 2.5 fold is ~65% of this value, which is in good agreement with the reduction in initiation to 62% as determined by hybrid selection NRO (Figure S3). We therefore conclude that transcription interference effects do not account for the increase in gene expression that is associated with termination. Quantitation shows relative mRNA levels where the AΔTERM value is 1. No pre-mRNA transcripts were detected beyond the linearisation site, showing that the templates remain linear in vivo (data not shown).

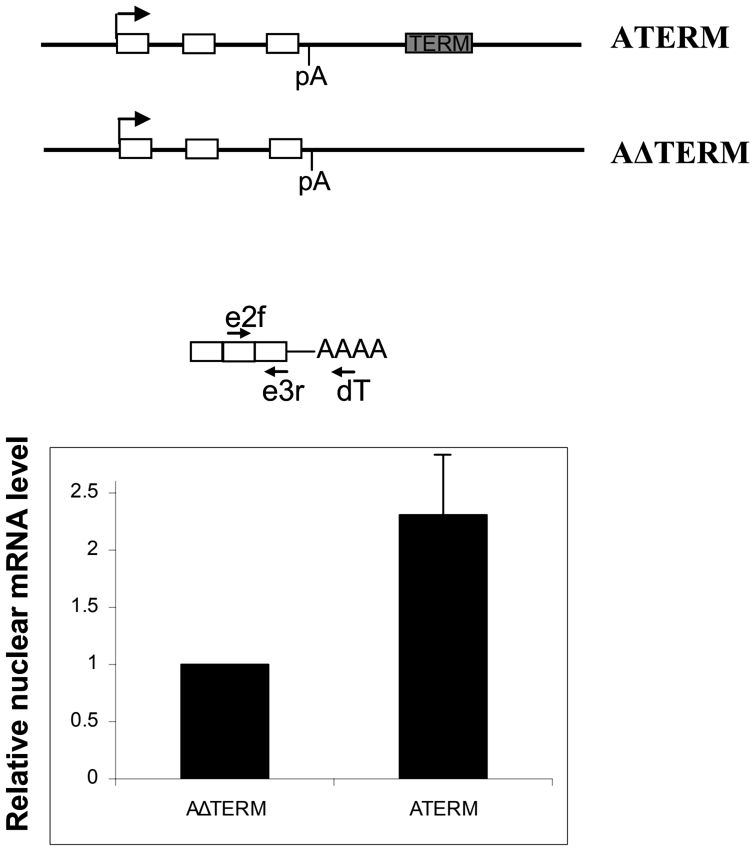

Figure S3: Further analysis of transcriptional interference

Transcriptional interference was quantitated using the assay described in Figure 1D. Nomenclature is also the same. Analysis was performed on βMAZ4, AΔTERM, PMΔTERM and EΔTERM. For βMAZ4 transcriptional interference was minimal because termination is efficient. In the other cases, initiation was reduced to between 60 and 70%, which is not sufficient to account for the large reduction in protein and mRNA levels observed in Figure 3. Note, that the addition of the terminator does not affect active Pol II density (Figure 1D).

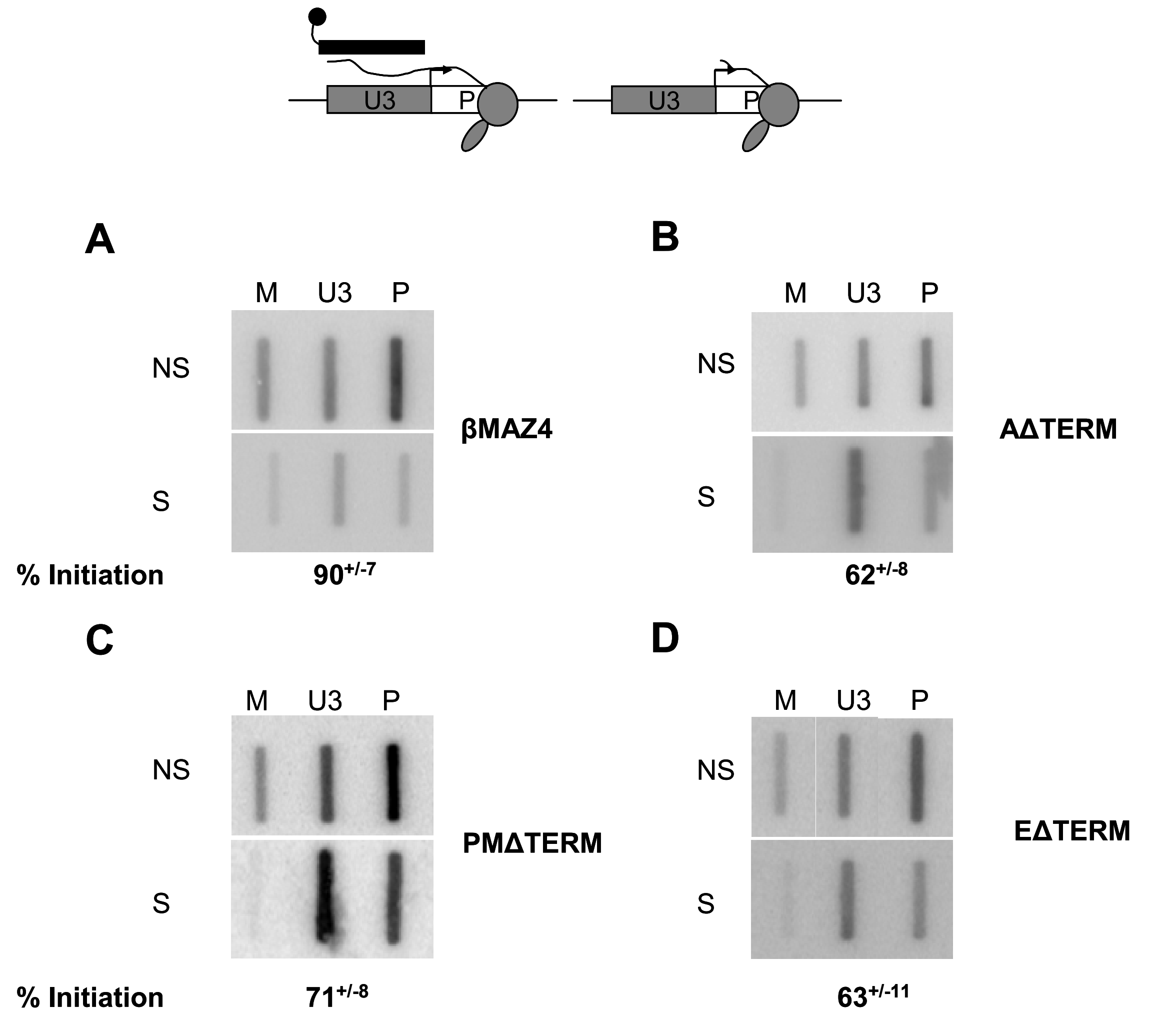

Figure S4: Promoter effects of co-transcriptional splicing.
BrU NRO was performed on HeLa cells transfected with βTERM or CβTERM and nascent RNA was analysed by RT-PCR to detect spliced exons 2 and 3. As such, RNA was reverse transcribed with primer e3r and subsequently amplified by real-time PCR using the e2f/e3r primer pair (see diagram). Although this primer pair may also detect unspliced transcripts, containing intron 2, this product is too long to be detected in our real-time PCR assay (data not shown). Only background levels (equal amount in parallel plus and minus antibody samples) of this species were detected in βTERM samples indicating that co-transcriptional splicing of exons 2 and 3 may not occur on this construct. In contrast, unspliced intron 2 can be detected (Figure 5D). Above background levels of spliced RNA was recovered from the CβTERM samples showing that co-transcriptional splicing occurs on this construct. The co-transcriptional splicing that is observed when transcription is from the CMV promoter is consistent with slower elongation, which may give the process more time to occur whilst Pol II is engaged with the DNA. This above-background signal also shows that intron 2 splicing can be observed with this assay.

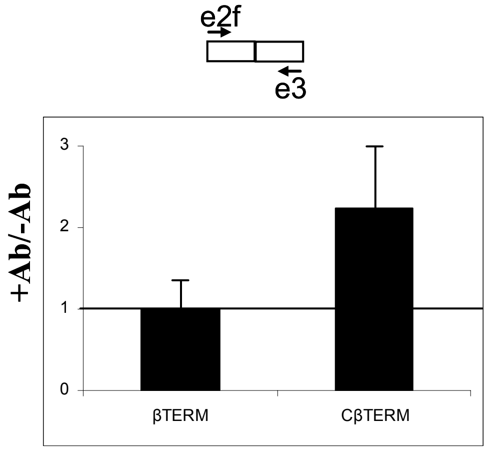

Supplement: Document S1. Four Figures and One Table [file mmc1.pdf]
